# Vascular abnormalities and development of hypoxia in microscopic melanoma xenografts

**DOI:** 10.1186/s12967-017-1347-9

**Published:** 2017-11-28

**Authors:** Jon-Vidar Gaustad, Trude G. Simonsen, Lise Mari K. Andersen, Einar K. Rofstad

**Affiliations:** Group of Radiation Biology and Tumor Physiology, Department of Radiation Biology, Institute for Cancer Research, Oslo University Hospital, Montebello, 0310 Oslo, Norway

**Keywords:** Microscopic tumors, Malignant melanoma, Vessel density, Vascular function, Vascular pericyte coverage, Tumor hypoxia, Angiogenic profiles

## Abstract

**Background:**

Studies investigating the oxygenation status and the development of hypoxia in microscopic tumors are sparse. The purpose of this study was to measure the extent of hypoxia in microscopic melanoma xenografts and to search for possible mechanisms leading to the development of hypoxia in these tumors.

**Methods:**

A-07, D-12, R-18, and U-25 human melanoma xenografts grown in dorsal window chambers or as flank tumors were used as preclinical tumor models. Morphologic and functional parameters of vascular networks were assessed with intravital microscopy, and the expression of angiogenesis-related genes was assessed with quantitative PCR. Microvessels, pericytes, and the extent of hypoxia were assessed by immunohistochemistry in microscopic tumors by using CD31, αSMA, and pimonidazole as markers, and the extent of radiobiological hypoxia was assessed in macroscopic flank tumors.

**Results:**

Macroscopic R-18 and U-25 tumors showed extensive hypoxia, whereas macroscopic A-07 and D-12 tumors were less hypoxic. R-18 and U-25 tumors developed hypoxic regions before they reached a size of 2–3 mm in diameter, whereas A-07 and D-12 tumors of similar size did not show hypoxic regions. The development of hypoxic regions was not caused by low vessel density, but was rather a result of inadequate vascular function. Inadequate vascular function was not caused by low vessel diameters or long vessel segments, but was associated with poor vascular pericyte coverage. Poor pericyte coverage was associated with the expression of eight angiogenesis-related genes.

**Conclusions:**

Two of the four investigated melanoma models developed hypoxic regions in microscopic tumors, and the development of hypoxia was associated with poor vascular pericyte coverage and inadequate vascular function.

## Background

Most tumors are heterogeneous in oxygen tension and develop regions with hypoxic cells during growth [1, 2]. Preclinical studies have demonstrated that tumors with extensive hypoxic regions are resistant to several types of therapy including radiation therapy, immunotherapy, and some forms of chemotherapy, and that tumor hypoxia may promote malignant progression and metastatic dissemination [1–4]. Clinical studies have shown that patients with highly hypoxic tumors have increased frequency of locoregional treatment failure, increased incidence of distant metastases and poor disease-free and overall survival rates following radiation therapy alone or radiation therapy combined with surgery and/or chemotherapy [1].

Tumor hypoxia is a consequence of inadequate oxygen supply caused by extensive abnormalities in the vascular network [2]. The vascular abnormalities include low and heterogeneous vessel density, elongated and tortuous vessels, aberrant vessel diameters, and vessel wall abnormalities (i.e., incomplete endothelial lining, interrupted basement membrane, and lack of pericytes and contractile vessel wall components) [2, 5, 6]. These morphological abnormalities collectively increase the geometric resistance to blood flow and impair vascular function [7]. Studies of macroscopic experimental tumors have shown that the severity of the vascular abnormalities increases during tumor growth, resulting in reduced blood supply and increased extent of hypoxia [8].

Studies investigating the development of hypoxia in microscopic tumors are sparse, but the extent of hypoxia has been measured in microscopic HT29 and HCT-8 colorectal adenocarcinoma xenografts [9]. In these models, tumors with diameters less than 1 mm were avascular and highly hypoxic, whereas tumors with diameters of 1–4 mm were vascularized and did not show hypoxic regions. The study suggested that clinical micrometastases may be resistant to ionizing radiation and chemotherapy before they induce angiogenesis, and that vascularized micrometastases may not show hypoxia-induced treatment resistance.

In the current study, we investigated the extent of hypoxia in microscopic A-07, D-12, R-18, and U-25 melanoma xenografts growing in dorsal window chambers. To search for mechanisms for the development of hypoxia, the morphology and function of the tumor vascular networks were studied with intravital microscopy and immunohistochemistry, and the expression of several angiogenesis-related genes was measured with quantitative PCR. The hypoxic status of microscopic tumors was compared with the extent of hypoxia in macroscopic tumors of the same melanoma models.

## Methods

### Tumor models

A-07, D-12, R-18, and U-25 human melanoma cells [10] were constitutively transfected with green fluorescence protein by lipofection, and the transfected cells used in this study were obtained from our frozen stock. Window chambers were surgically implanted in the dorsal skin fold of adult female BALB/c *nu/nu* mice as reported earlier [11]. Tumors were initiated by implanting multicellular spheroids or solid tumor pieces in window chambers, or by inoculating approximately 3.5 × 10^5^ cells intradermally in the mouse flank. Female mice were used for practical reasons.

### Anesthesia

Window chamber implantation and intravital microscopy examinations were carried out with anesthetized mice. Fentanyl citrate (Janssen Pharmaceutica, Beerse, Belgium), fluanisone (Janssen Pharmaceutica), and midazolam (Hoffmann-La Roche, Basel, Switzerland) were administered intraperitoneally in doses of 0.63, 20, and 10 mg/kg, respectively.

### Intravital microscopy

Mice with window chambers were fixed to the microscope stage during intravital microscopy, and the body core temperature was kept at 37–38 °C by using a hot-air generator. Imaging was performed by using an inverted fluorescence microscope (IX-71; Olympus, Munich, Germany) and a black and white CCD camera (C9300-024; Hamamatsu Photonics, Hamamatsu, Japan). Tumor vasculature was visualized by using a ×4 objective lens, transillumination, and filters for green light, resulting in images with a pixel size of 3.7 × 3.7 μm^2^. Vessel density (i.e., total vessel length per mm^2^ tumor area), median intercapillary distance (i.e., the distance from a tumor vessel to the nearest neighboring vessel), and median vessel diameter were computed from manually produced vascular masks of the entire vascular networks, as previously described [11]. Median vessel segment length was calculated from ~ 50 randomly selected vessel segments. To investigate vascular function, first-pass imaging movies were recorded after a 0.2 mL bolus of 50 mg/mL tetramethylrhodamine isothiocyanate-labeled dextran (Sigma-Aldrich, Schnelldorf, Germany) with a molecular weight of 155 kDa was injected into the lateral tail vein. First-pass imaging movies were recorded at a rate of 22.3 frames per second by using a 2× objective lens, resulting in a time resolution of 44.8 ms and a pixel size of 7.5 × 7.5 μm^2^. Blood supply time (BST) images were produced by assigning a BST value to each pixel of the vascular masks [11]. The BST of a pixel was defined as the time difference between the frame showing maximum fluorescence intensity in the pixel and the frame showing maximum fluorescence intensity in the main tumor supplying artery, as described in detail previously [12].

### Immunohistochemical detection of tumor hypoxia, microvessels, and pericytes

The tumors were resected immediately after the intravital microscopy examinations and fixed in phosphate-buffered 4% paraformaldehyde. Pimonidazole [1-[(2-hydroxy-3-piperidinyl)-propyl]-2-nitroimidazole], administered as described previously [13], was used as a hypoxia marker, CD31 was used as a marker for endothelial cells, and α-smooth muscle actin (αSMA) was used as a marker for pericytes. Immunohistochemistry was done by using a peroxidase-based indirect staining method [13]. An anti-pimonidazole rabbit polyclonal antibody (gift from Prof. J.A. Raleigh, Department of Radiation Oncology, University of North Carolina School of Medicine, Chapel Hill, NC), an anti-CD31 rabbit polyclonal antibody (Ab28364; Abcam, Cambridge, United Kingdom), or an anti-αSMA rabbit polyclonal antibody (Ab5694; Abcam) was used as primary antibody. Diaminobenzidine was used as chromogen, and hematoxylin was used for counterstaining. Hypoxic area fractions were determined by image analysis. Number of CD31-positive and αSMA-positive profiles per mm^2^ of tumor tissue (#/mm^2^) was scored manually.

### Radiobiological assessment of tumor hypoxia

The paired survival curve method was used to measure radiobiological hypoxia [13]. Tumors were irradiated under air-breathing or hypoxic conditions at a dose rate of 5.1 Gy/min by using an X-ray unit operated at 220 kV, 19–20 mA, and with 0.5 mm Cu filtration. Hypoxic conditions were obtained by occluding the tumor blood supply with a clamp 5 min before irradiation. Tumor cell survival was measured in vitro. The tumors were resected immediately after irradiation, minced in cold Hanks’ balanced salt solution, and treated with an enzyme solution (0.2% collagenase, 0.05% pronase, and 0.02% DNase) at 37 °C for 2 h. Trypan blue-negative cells were plated in 25 cm^2^ tissue culture flasks and incubated at 37 °C for 14 days for colony formation. The cell surviving fraction of an irradiated tumor was calculated from the number of cells plated, the number of colonies counted, and the mean plating efficiency of six untreated control tumors. Cell survival curves were established for clamped and unclamped tumors, and the fraction of radiobiologically hypoxic cells (HF_RAD_) was calculated from the vertical displacement of the curves [13].

### Quantitative PCR

RNA isolation, cDNA synthesis, and quantitative PCR were performed as described in detail previously [14]. Briefly, gene expression was assessed by using the RT^2^ Profiler PCR Array Human Angiogenesis (PAHS-024A) from SABiosciences (Frederick, MD). Real-time PCR was performed on an ABI 7900HT Fast Real-Time PCR instrument (Applied Biosystems, Carlsbad, CA). Each tumor line was run in three biological replicates. Glyceraldehyde-3-phosphate dehydrogenase (GAPDH) and β-actin (ACTB) were used as normalization genes because these housekeeping genes showed stable expression across the melanoma lines studied here. Thus, each replicate C_T_ value was normalized to the mean C_T_ value of GAPDH and ACTB (ΔC_T_  =  C_T_^gene of interest ^− C_T_^mean of GADPH and ACTB^). The normalized expression level of each gene was calculated from the three biological replicates as 2^−meanΔCT^.

### Statistical analysis

Statistical comparisons of data were performed by one-way analysis of variance followed by the Student–Neuman–Keuls test when the data complied with the conditions of normality and equal variance, and under other conditions by the Kruskal–Wallis one-way analysis of variance on ranks followed by the Dunn’s test. Probability values of *P* < 0.05, determined from two-sided tests, were considered significant. The statistical analysis was performed by using the SigmaStat statistical software (SPSS Science, Chicago, IL).

## Results

**Fig. 1:**
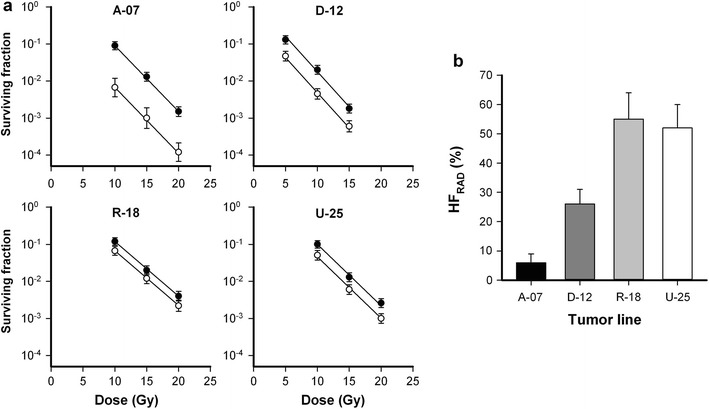
Hypoxia in macroscopic tumors. **a** Cell survival curves of ~ 400 mm^3^ flank tumors irradiated under air breathing (open circle) or hypoxic (filled circle) conditions. Points, geometric mean of 5–7 tumors; bars, SE. **b** Fraction of radiobiologically hypoxic cells (HF_RAD_) in A-07, D-12, R-18, and U-25 tumors. HF_RAD_ was calculated from the survival curves in **a**. Columns, mean values; bars, SE

HF_RAD_ was measured in macroscopic flank tumors (~ 400 mm^3^) by using the paired survival curve method. Figure 1a shows paired survival curves for A-07, D-12, R-18, and U-25 melanoma xenografts. HF_RAD_ was significantly higher in R-18 and U-25 tumors than in A-07 and D-12 tumors, and significantly higher in D-12 tumors than in A-07 tumors (Fig. 1b; *P* < 0.01).

**Fig. 2:**
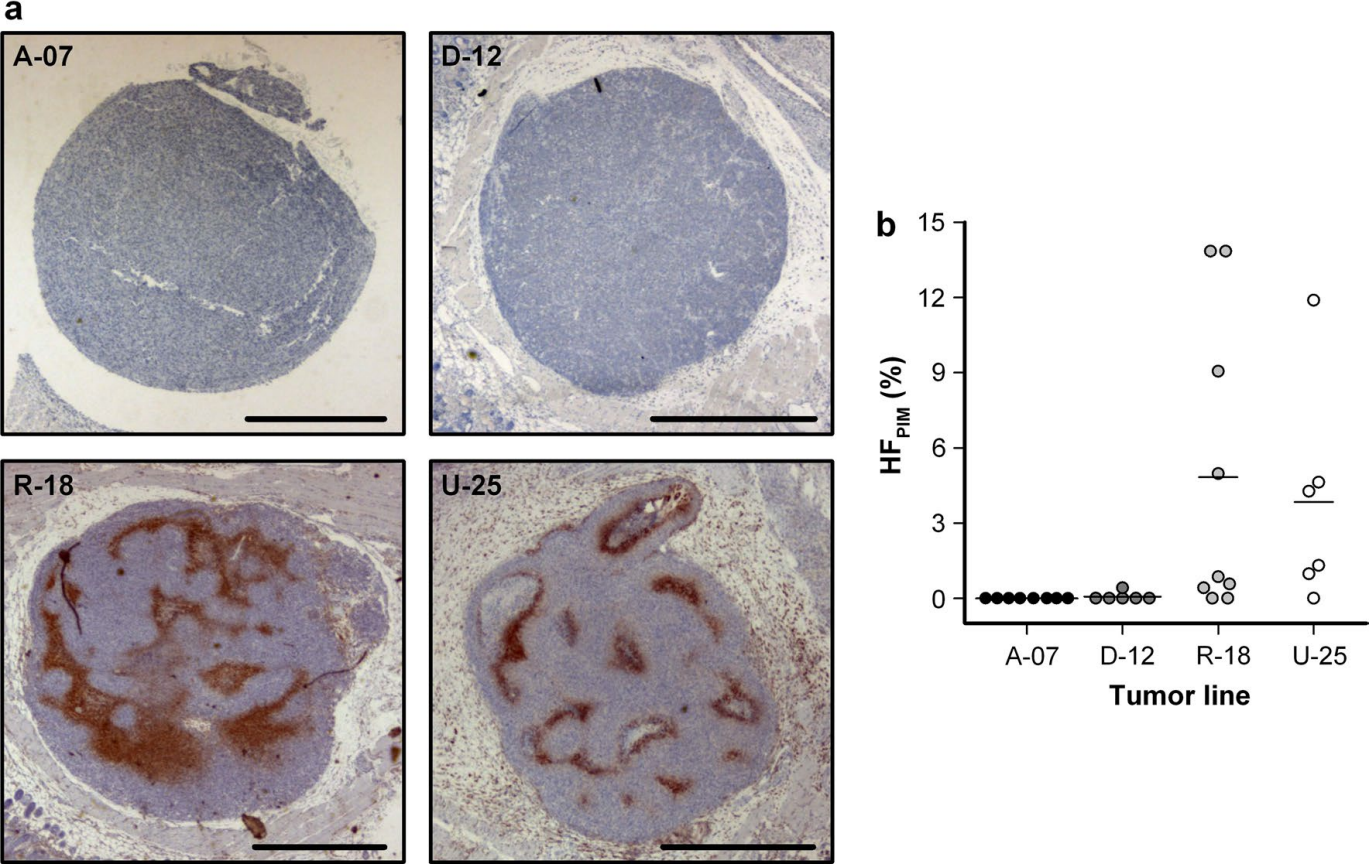
Hypoxia in microscopic tumors. **a** Immunohistochemical preparations of representative microscopic A-07, D-12, R-18, and U-25 window chamber tumors stained with pimonidazole to visualize hypoxia. Scale bars, 1 mm. **b** Fraction of pimonidazole-positive cells (HF_PIM_) in microscopic A-07, D-12, R-18, and U-25 window chamber tumors. Points, individual tumors; horizontal lines, means

To investigate the development of hypoxia in microscopic tumors, A-07, D-12, R-18, and U-25 tumors were initiated in dorsal window chambers and subjected to histological examination. Figure 2a shows immunohistochemical preparations of representative A-07, D-12, R-18, and U-25 tumors stained for pimonidazole. 78% of the R-18 tumors and 83% of the U-25 tumors had developed hypoxic regions before they reached a size of 2–3 mm in diameter, whereas A-07 and D-12 tumors with similar sizes did not show hypoxic regions (Fig. 2). The fraction of pimonidazole-positive cells (HF_PIM_) was thus significantly higher in R-18 and U-25 tumors than in A-07 and D-12 tumors (Fig. 2b; *P* < 0.01).

**Fig. 3:**
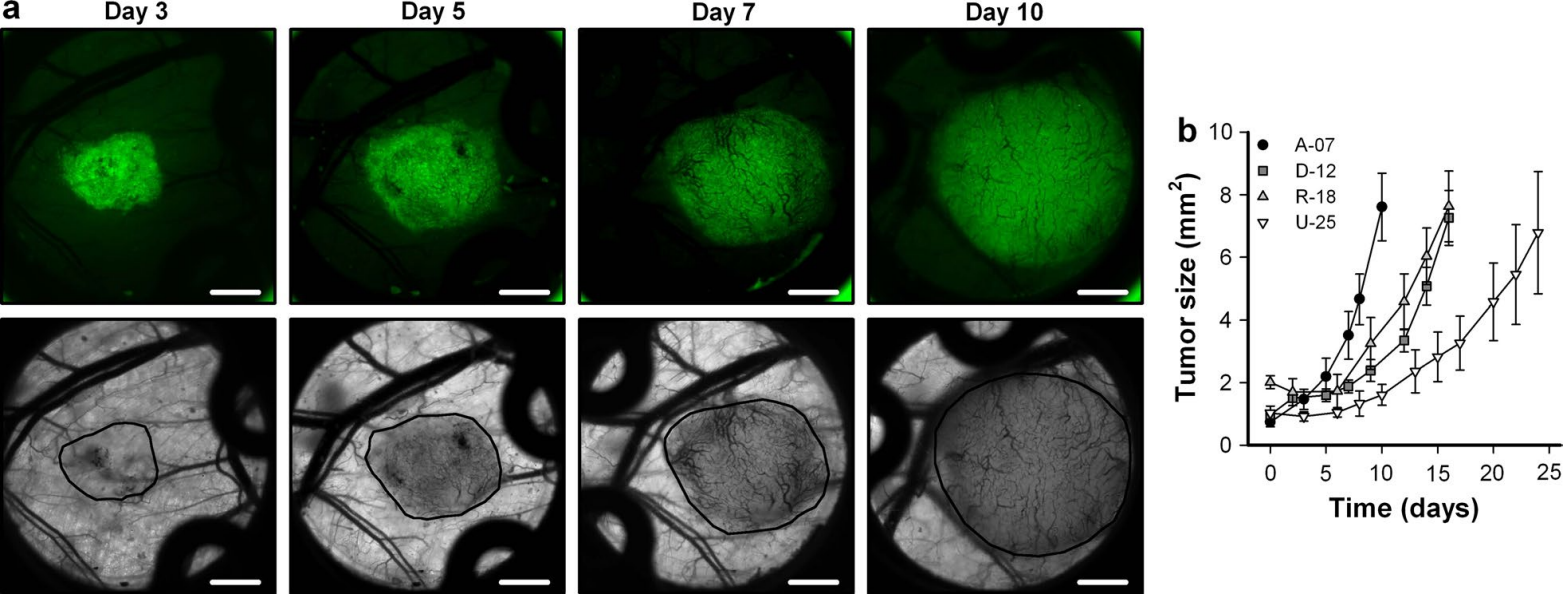
Tumor growth and vascularization. **a** Intravital microscopy images of a representative microscopic A-07 window chamber tumor recorded 3, 5, 7 and 10 days after tumor initiation. The panels show fluorescence images of the GFP-expressing tumor cells (upper row) and transillumination images of the vasculature (lower row). Tumor area is delineated by a black line in the transillumination images. Scale bars, 1 mm. **b** Tumor area versus time for microscopic A-07, D-12, R-18, and U-25 window chamber tumors. Points, means of 6–13 tumors; bars, SE

The microscopic tumors were subjected to intravital microscopy multiple times during growth. Figure 3a shows intravital microscopy images of a representative A-07 tumor, and illustrates the first signs of angiogenesis (small bleedings within the tumor mass; day 3), novel tumor vessels (day 5), and an established vascular network (day 7 and day 10). In D-12, R-18, and U-25 tumors, the first tumor vessels appeared later, and the tumor growth rates were significantly lower than in A-07 tumors (Fig. 3b; *P* < 0.05). U-25 tumors showed lower growth rates than D-12 and R-18 tumors (*P* < 0.05), whereas the hypoxia-positive R-18 tumors did not differ from the hypoxia-negative D-12 tumors in growth rate (*P* > 0.05).

**Fig. 4:**
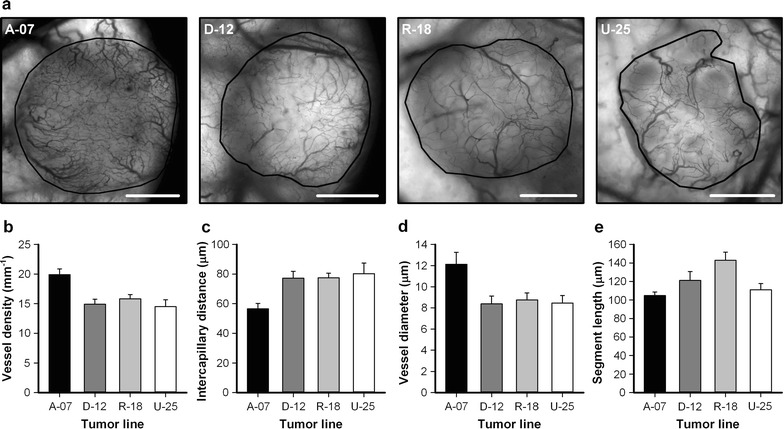
Vascular morphology. **a** Intravital microscopy images of representative microscopic A-07, D-12, R-18, and U-25 window chamber tumors. Tumor area is delineated by a black line. Scale bars, 1 mm. **b**–**e** Vessel density (**b**), median intercapillary distance (**c**), median vessel diameter (**d**), and median vessel segment length (**e**) in microscopic A-07, D-12, R-18, and U-25 window chamber tumors. Columns, means of 6–13 tumors; bars, SE

The melanoma models were allowed different growth times before they were resected for histological examination in accordance with the differences in tumor growth rate (Fig. 3b). Figure 4a shows high resolution intravital microscopy images of the vasculature of representative A-07, D-12, R-18, and U-25 tumors immediately before the tumors were resected. At this time point, all tumors were highly vascularized. The vessel segments were uniformly distributed, and avascular regions were not observed in any of the tumors. A-07 tumors showed higher vessel density and lower intercapillary distance than D-12, R-18, and U-25 tumors (Fig. 4b, c; *P* < 0.05). A-07 tumors also showed larger vessel diameters than D-12, R-18, and U-25 tumors (Fig. 4d; *P* < 0.05), whereas R-18 tumors showed longer vessel segments than A-07 and U-25 tumors (Fig. 4e; *P* < 0.05). None of these morphological parameters distinguished between the hypoxia-positive (R-18 and U-25) and the hypoxia-negative tumors (A-07 and D-12).

**Fig. 5:**
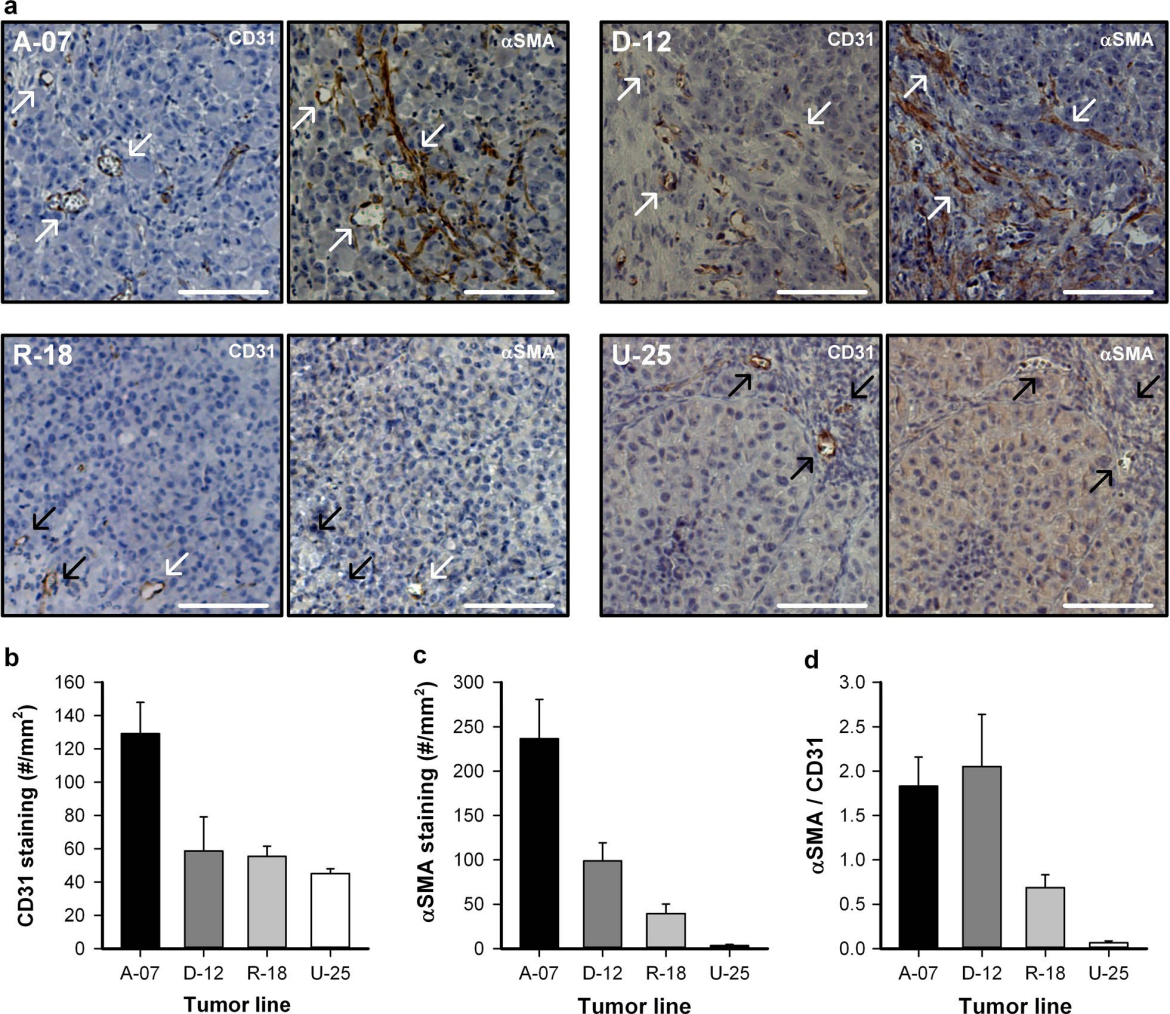
Immunohistological assessment of microvessels and pericytes. **a** Adjacent immunohistological sections of representative microscopic A-07, D-12, R-18, and U-25 window chamber tumors stained with CD31 to visualize microvessels or αSMA to visualize pericytes. Examples of CD31 positive vessels with or without αSMA staining are highlighted with white or black arrows, respectively. Scale bars, 100 μm. **b**–**d** Density of CD31 positive profiles (**b**), density of αSMA positive profiles (**c**), and the ratio between αSMA and CD31 positive profiles (**d**)

Adjacent histological tumor sections were stained with CD31 to visualize microvessels or with αSMA to visualize pericytes. CD31 positive vessels with or without positive αSMA staining are highlighted in Fig. 5a with white or black arrows, respectively. In A-07 and D-12 tumors, most CD31 positive vessels showed positive αSMA staining, and long bands with positive αSMA staining were observed between microvessels. In contrast, only a few CD31 positive vessels showed positive αSMA staining in R-18 and U-25 tumors, and positive αSMA staining were only found adjacent to microvessels. In agreement with the intravital microscopy observations, the density of CD31 positive vessels was higher in A-07 tumors than in D-12, R-18, and U-25 tumors (Fig. 5b; *P* < 0.05). The density of αSMA positive profiles was higher in A-07 tumors than in D-12, R-18, and U-25 tumors, and higher in D-12 tumors than in R-18 and U-25 tumors (Fig. 5c; *P* < 0.05). Moreover, the ratio between αSMA and CD31 positive profiles was higher for A-07 and D-12 tumors than for R-18 and U-25 tumors (Fig. 5d; *P* < 0.05), and did not differ between A-07 and D-12 tumors (Fig. 5d; *P* > 0.05).

**Fig. 6:**
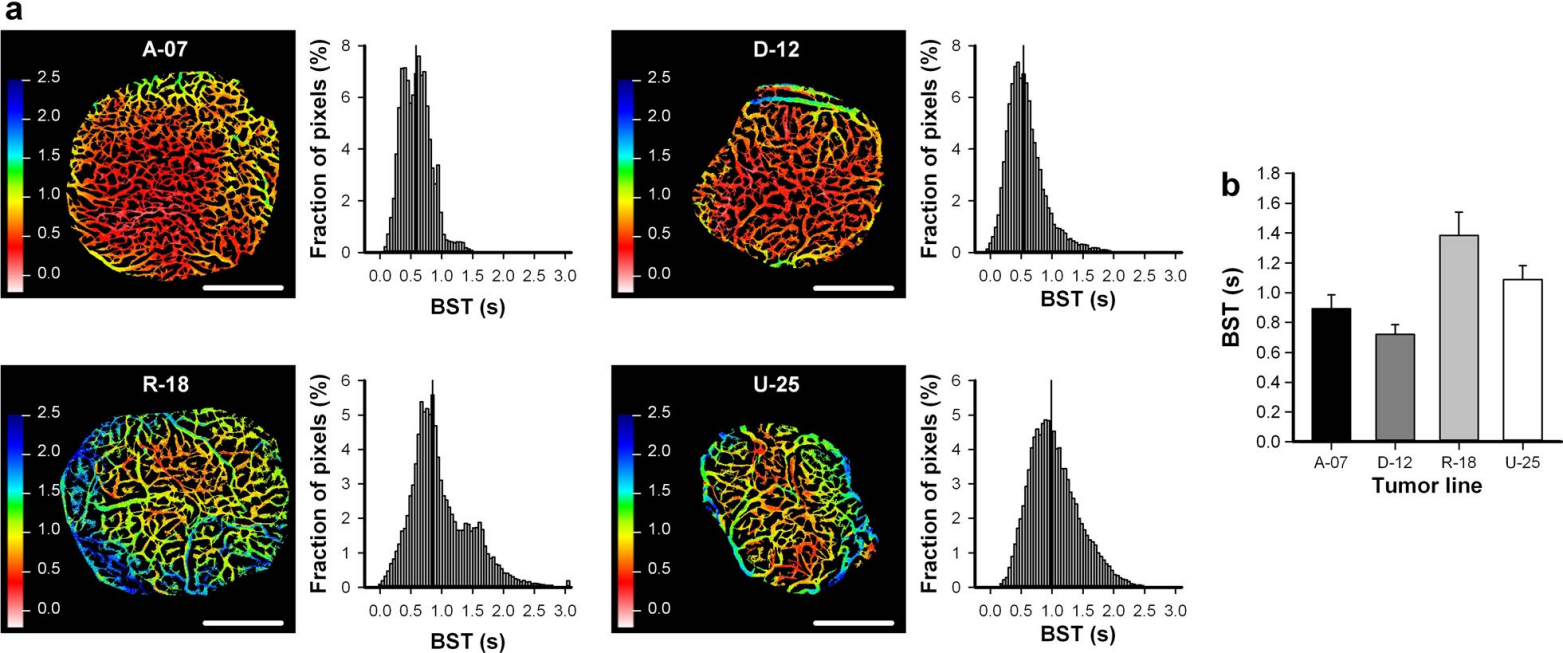
Vascular function. **a** Blood supply time (BST) images and the corresponding BST frequency distributions of representative microscopic A-07, D-12, R-18, and U-25 window chamber tumors. Color bars, BST scales in seconds; scale bars, 1 mm. **b** Median BST in microscopic A-07, D-12, R-18, and U-25 window chamber tumors. Columns, means of 7–17 tumors; bars, SE

To investigate the function of the vascular networks, first-pass imaging movies were recorded and BST images and BST frequency distributions were produced. The BST image and the BST frequency distribution of representative A-07, D-12, R-18, and U-25 tumors are shown in Fig. 6a. D-12 tumors showed lower BST values than R-18 and U-25 tumors (Fig. 6b; D-12 vs R-18: *P* < 0.01; D-12 vs U-25: *P* = 0.012), and A-07 tumors showed lower BST values than R-18 tumors (Fig. 6b; *P* < 0.01).

**Fig. 7:**
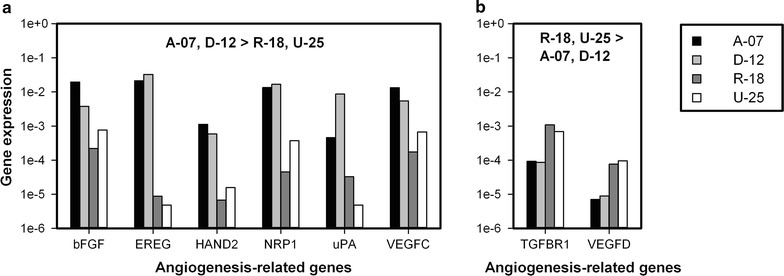
Expression of angiogenesis-related genes. Normalized expression (2^−ΔCT^) of angiogenesis-related genes that are more than fivefold higher expressed in the A-07 and D-12 models than in the R-18 and U-25 models (**a**), and more than fivefold higher expressed in the R-18 and U-25 models than in the A-07 and D-12 models (**b**). Gene expression was measured with quantitative PCR and normalized to housekeeping genes with stable expression (GAPDH and ACTB)

The expression of 84 known angiogenesis-related genes was quantified by using a commercial quantitative PCR array. The genes encoding basic fibroblast growth factor (bFGF), epiregulin (EREG), basic helix-loop-helix transcription factor (HAND2), neuropilin 1 (NRP1), urokinase-type plasminogen activator (uPA), and vascular endothelial growth factor C (VEGFC) showed higher expression (> fivefold) in the A-07 and D-12 models than in the R-18 and U-25 models (Fig. 7a), whereas the genes encoding transforming growth factor β receptor 1 (TGFBR1) and VEGFD showed higher expression (> fivefold) in the R-18 and U-25 models than in the A-07 and D-12 models (Fig. 7b).

## Discussion

Tumor hypoxia was assessed by using a radiobiological assay in macroscopic tumors. This assay measures the fraction of the clonogenic cells in tumors that are hypoxic [13]. HF_RAD_ is considered to be of great clinical relevance, because only the clonogenic cells are relevant for tumor growth and response to treatment [15]. Unfortunately, HF_RAD_ cannot be measured without severe problems in microscopic tumors, and consequently an immunohistochemical assay using pimonidazole as hypoxia marker was used to detect hypoxia in window chamber tumors. The immunohistochemical assay provides information of the spatial distribution of hypoxia, but does not distinguish between clonogenic and non-clonogenic cells [13]. The absolute values of HF_RAD_ were substantially higher than the absolute values of HF_PIM_. These differences probably reflected the different cell types that were measured with the assays (clonogenic versus all cells) and were also influenced by the differences in tumor size (macroscopic versus microscopic tumors). In addition, HF_RAD_ is sensitive to both chronically and acutely hypoxic cells whereas the immunohistochemical assay has been optimized to preferentially stain chronically hypoxic cells [13]. Despite these differences, the melanoma models that developed hypoxia in microscopic tumors (R-18 and U-25) also showed the highest HF_RAD_ in macroscopic tumors. This observation suggests that hypoxia was caused by tumor-line specific abnormalities, but does not imply that the hypoxic status of tumors is static. Thus it is well known that the extent of tumor hypoxia can increase during growth and may be altered by treatments [8]. We have previously shown that subcurative radiation and antiangiogenic treatment can increase the hypoxic fractions in the melanoma models included in the current study [16, 17].

R-18 and U-25 tumors developed hypoxic regions before they reached a size of 2–3 mm in diameter, whereas A-07 and D-12 tumors of similar sizes did not show hypoxic regions. Hypoxia was not caused by low vessel densities in microscopic tumors because all the tumors were highly vascularized, and the hypoxic R-18 and U-25 tumors showed similar vessel densities as the non-hypoxic D-12 tumors. By using typical values for intracapillary oxygen tensions and tumor tissue oxygen consumption rates, the oxygen diffusion distance has been calculated to be approximately 150 μm in tumor tissue [2]. These theoretical calculations imply that tumor hypoxia and necrosis can be expected if the intercapillary distance exceeds 300 μm. The intercapillary distances measured with intravital microscopy were low compared to this critical limit. However, intravital microscopy images show 2-dimensional projections of the 3-dimensional vasculature, and are expected to underestimate intercapillary distances [18]. Intercapillary distances were also calculated from histological sections stained for microvessels by assuming a homogenous vessel distribution. These distances ranged from ~ 100 μm (A-07) to ~ 150 μm (D-12, R-18, and U-25) and were thus twofold to threefold lower than the critical limit determined by the oxygen diffusion distance [2].

Mathematical simulations and experimental studies have demonstrated that hypoxic regions may develop close to blood vessels if the vascular function is impaired [2, 7, 19]. The hypoxic R-18 and U-25 tumors showed higher BST values and hence lower blood flow velocities than the non-hypoxic A-07 and D-12 tumors, suggesting that development of hypoxia was a result of inadequate vascular function. Narrow and elongated vessels may reduce blood flow due to high geometric resistance [7]. Large vessel diameters may thus have contributed to efficient blood supply in A-07 tumors, and narrow and elongated vessels may have contributed to poor vascular function in R-18 tumors. However, the non-hypoxic D-12 tumors did not differ from hypoxic U-25 tumors in vessel diameter or vessel segment length, suggesting that other parameters were more important in governing vascular function in these tumors.

Pericytes are known to provide structural support to blood vessels and to influence vessel stability [20]. Moreover, depletion of pericytes in genetically engineered mouse models has been shown to impair vascular function and induce hypoxia in breast cancer xenografts [21]. In the present study, αSMA was used as a marker for pericytes. The majority of the vessels in A-07 and D-12 tumors showed positive αSMA staining whereas only a few vessels showed positive αSMA staining in R-18 and U-25 tumors. This observation implies that the melanoma models differed in vascular pericyte coverage, and poor pericyte coverage was associated with inadequate vascular function and development of hypoxia. This finding suggests that vascular pericyte coverage may have prognostic value in clinical melanomas, a suggestion that merits clinical investigation. In line with this suggestion, patients with no detectable vascular pericyte coverage had lower disease-free and overall survival rates than patients with detectable pericyte coverage in a study of 130 patients with invasive breast cancer [22]. As with all available pericyte markers, αSMA is also expressed by some other cell types, including smooth muscle, myofibroblasts, and myoepithelium [20]. It is thus possible that also other cell types were identified by αSMA staining in the present study.

The current study demonstrates that microscopic tumors with high vessel densities may develop regions with hypoxic tissue if the vascular function is inadequate. This finding differs from a study by Li et al. which reported that vascularized colorectal carcinoma xenografts with a diameter of 1–4 mm did not show hypoxic regions [9], but is in agreement with a study by Cao et al. which found evidence of hypoxia in vascularized colon carcinoma xenografts with a diameter of 1–2 mm [23]. The finding has important implications for clinical micrometastases because micrometastatic disease is often treated with chemotherapy or regional radiotherapy after the primary tumor has been surgically removed. If clinical micrometastases have developed regions with hypoxic tissue, they may be resistant to these treatments [2]. Clinical studies have suggested that the treatment of primary tumors may be improved by combining radiation therapy with hypoxia-modifying agents [24]. It is possible that also the treatment of micrometastatic disease may be improved by using similar strategies to reduce hypoxia-induced treatment resistance.

Several angiogenic stimulators have been shown to be important for initiation and maintenance of angiogenesis in melanoma, including VEGFA, interleukin-8 (IL-8), bFGF, and uPA [25]. In the present study, the A-07 and D-12 models showed higher expression of the genes encoding bFGF, EREG, HAND2, NRP1, uPA, and VEGFC, and lower expression of the genes encoding TGFBR1 and VEGFD than the R-18 and U-25 models. These genes have been reported to regulate angiogenesis, vascular remodeling, and/or lymphangiogenesis, but their role in regulating vascular function is not clear [25–31]. Moreover, these genes are not found among the genes reported to show frequent mutations in melanoma [32]. The expression of these genes was associated with vascular function, and the development of hypoxia in melanoma xenografts. However further studies are needed to determine whether there is a causal relationship between these parameters.

We have previously shown that angiogenesis can be inhibited by targeting the VEGFA pathway in A-07 tumors, and this treatment reduced vessel densities and induced hypoxia [33]. We have also demonstrated that artificially imposed hypoxia (i.e., by exposing tumor-bearing mice to a low oxygen atmosphere) can increase VEGFA expression and induce angiogenesis in the same tumor model [34]. These experiments illustrate the complex interplay between angiogenesis, vasculature, and hypoxia in tumors, and imply that several experiments with different experimental design may be needed to determine causal relationships.

Clinical investigations have revealed that low oxygen tension and hypoxic tumor regions are characteristic features of the physiological microenvironment of human melanomas [35, 36]. Various antiangiogenic drugs targeting the VEGFA pathway have been evaluated in clinical trials but none of the drugs have improved survival for patients with malignant melanoma [25]. The current study identifies other angiogenic stimulators than VEGFA that are associated with vascular function and the development of hypoxia in microscopic melanoma xenografts. It is possible that antiangiogenic drugs targeting these angiogenic pathways can be effective in human melanomas, and that such drugs can improve vascular function and tumor oxygenation. In line with this speculation, it has been demonstrated that low doses of antiangiogenic agents can improve vascular function and increase oxygenation in several experimental tumor models, including human ovarian carcinoma xenografts, human neuroblastoma xenografts, human glioma xenografts, murine melanoma, and murine breast carcinoma [37–39].

## Conclusions

Two of the four investigated melanoma xenograft models developed hypoxic regions before they reached a size of 2–3 mm in diameter. Poor vascular pericyte coverage and inadequate vascular function were associated with the development of hypoxia in microscopic tumors, and the vascular abnormalities were associated with the angiogenic profile of the melanoma xenograft models.

## Abbreviations

bFGF: basic fibroblast growth factor
BST: blood supply time
EREG: epiregulin
HAND2: basic helix-loop-helix transcription factor
HF_PIM_: fraction of pimonidazole-positive cells
HF_RAD_: fraction of radiobiologically hypoxic cells
NRP1: neuropilin 1
TGFBR1: transforming growth factor β receptor 1
uPA: urokinase-type plasminogen activator
VEGF: vascular endothelial growth factor
